# Avian Models for Human Carcinogenesis—Recent Findings from Molecular and Clinical Research

**DOI:** 10.3390/cells13211797

**Published:** 2024-10-30

**Authors:** Julia Niebora, Krzysztof Data, Dominika Domagała, Małgorzata Józkowiak, Saoirse Barrett, Tannaz Norizadeh Abbariki, Artur Bryja, Magdalena Kulus, Sławomir Woźniak, Hanna Ziemak, Hanna Piotrowska-Kempisty, Paweł Antosik, Dorota Bukowska, Paul Mozdziak, Piotr Dzięgiel, Bartosz Kempisty

**Affiliations:** 1 Division of Anatomy, Department of Human Morphology and Embryology, Faculty of Medicine, Wroclaw Medical University, 50-368 Wroclaw, Polanddominika.domagala@umw.edu.pl (D.D.); malgorzata.jozkowiak@umw.edu.pl (M.J.);; 2 Department of Toxicology, Poznan University of Medical Sciences, 60-631 Poznan, Poland; 3 Human Clinical Embryology & Assisted Conception, School of Medicine, University of Dundee, Dundee DD1 4HN, UK; 4 Flyblast BV, 2020 Antwerp, Belgium; 5 Department of Veterinary Surgery, Institute of Veterinary Medicine, Nicolaus Copernicus University in Torun, 87-100 Torun, Poland; 6 Veterinary Clinic of the Nicolaus Copernicus University in Torun, 87-100 Torun, Poland; 7 Department of Basic and Preclinical Science, Institute of Veterinary Medicine, Nicolaus Copernicus University in Torun, 87-100 Torun, Poland; 8 Department of Diagnostics and Clinical Sciences, Institute of Veterinary Medicine, Nicolaus Copernicus University in Torun, 87-100 Torun, Poland; 9 Prestage Department of Poultry Science, North Carolina State University, Raleigh, NC 27695, USA; pemozdzi@ncsu.edu; 10 Physiology Graduate Faculty, North Carolina State University, Raleigh, NC 27695, USA; 11 Division of Histology and Embryology, Department of Human Morphology and Embryology, Faculty of Medicine, Wroclaw Medical University, 50-367 Wroclaw, Poland; 12 Center of Assisted Reproduction, Department of Obstetrics and Gynecology, University Hospital and Masaryk University, 601 77 Brno, Czech Republic

**Keywords:** oncoviruses, hen model, ovarian cancer, carcinogenesis, chorioallantoic membrane

## Abstract

Birds, especially the chick and hen, have been important biomedical research models for centuries due to the accessibility of the avian embryo and the early discovery of avian viruses. Comprehension of avian tumor virology was a milestone in basic cancer research, as was that of non-viral genesis, as it enabled the discovery of oncogenes. Furthermore, studies on avian viruses provided initial insights into Kaposi’s sarcoma and EBV-induced diseases. However, the role of birds in human carcinogenesis extends beyond the realm of virology research. Utilization of CAM, the chorioallantoic membrane, an easily accessible extraembryonic tissue with rich vasculature, has enabled studies on tumor-induced angiogenesis and metastasis and the efficient screening of potential anti-cancer compounds. Also, the chick embryo alone is an effective preclinical in vivo patient-derived xenograft model, which is important for the development of personalized therapies. Furthermore, adult birds may also closely resemble human oncogenesis, as evidenced by the laying hen, which is the only animal model of a spontaneous form of ovarian cancer. Avian models may create an interesting alternative compared with mammalian models, enabling the creation of a relatively cost-effective and easy-to-maintain platform to address key questions in cancer biology.

## 1. Introduction

Cancer is a leading cause of death worldwide. In 2018, there were over 18.1 million new cases and 9.5 million cancer-related deaths, and as of 2019, about 18 million new cases occurred annually. By 2040, it is estimated that the number of new cancer cases per year will rise to 29.5 million and the number of cancer-related deaths to 16.4 million [1]. Cancer presents an immense burden and strain to individuals and their loved ones as well as to healthcare systems.

Cancer formation represents a complex, multistep process. In recent decades, it has become obvious that viruses play a significant role in the multistage development of human tumors. About 15% to 20% of cancers are related to viral infections. Oncogenic viruses play a role in different steps of the cancer-causing process, and the association of a virus with a given cancer can be anywhere from 15% to 100% [2].

Avian retroviruses were first identified as cancer-causing filtering agents in chicken tumors in the early 20th century. However, avian retroviruses have continued to evolve since their discovery, and it is worth focusing further research, as studying these viruses has contributed to a better understanding of the mechanisms of viral replication and cancer [3].

## 2. Avian Retroviruses

Avian retroviruses are a group of RNA viruses that mainly infect birds and can integrate their genetic material into the host cell’s DNA. Avian retroviruses are known to cause various diseases in birds, including cancer, immunosuppression, and other disorders [4]. The most prevalent naturally occurring avian retroviruses are avian leukosis virus (ALV), reticuloendotheliosis virus (REV), and lymphoproliferative disease virus (LPDV). ALV is an alpharetrovirus associated with cancer, growth retardation, and reduced fertility. It should also be noted that ALV causes severe immunosuppression, increasing susceptibility to other microbial infections and the risk of failure of subsequent vaccinations against other diseases [5]. REV is a retrovirus that mainly infects birds, including chickens, turkeys, and ducks. It belongs to the genus gammaretrovirus in the family *Retroviridae*. REV can cause a variety of clinical signs in infected birds, including immunosuppression, neoplastic disease (tumors), and oncogenic anemia. LPDV is a rare, poorly understood oncogenic retrovirus of domestic turkeys that also belongs to the *Retroviridae* family. The characteristic feature of lymphoproliferative disease is the infiltration of pleomorphic lymphoid cells into multiple organs and tissues, which, if proliferation is uncontrolled, can contribute to lymphosarcoma. The natural routes of transmission of LPDV are not well understood [6].

### 2.1. Reticuloendotheliosis Virus and Avian Leukosis Virus

REV and ALV are simple retroviruses that separately represent significant oncogenic agents with variable epidemiology, especially depending on region and flock specificity. They are found in the wild as well as in poultry farming [7,8,9]. While ALV epidemiology is limited to gallinaceous birds [10], REV can infect a wide range of bird species, including chickens, pigeons, turkeys, ducks, and geese [8,11]. Although both viruses belong to the retroviruses, REV-induced cancers differ from ALV-induced cancers such as induced lymphomas. B-cell lymphoma is the most common ALV-induced neoplasm [12]. In contrast, REV-induced B-cell lymphoma is rare and is associated with prolonged viral latency, and it depends on insertional mutagenesis as a tumor inducer [13].

Growth retardation syndrome usually manifests as weight loss, pallor, and plumage abnormalities (also Nakanuke disease), but the role of REV in its pathogenesis is still not well characterized [14]. A member of the REV group of viruses is the T strain of reticuloendotheliosis virus (REV-T). It was first isolated in the lesions of a turkey that died of visceral reticuloendotheliosis (RE) in 1957 [15]. While other REVs are pathogenic, REV-T is unique in its role as an acute transformant, exhibiting the capacity for rapid transformation of primary avian hematopoietic cells. This is due to the acquisition of the avian reticuloendotheliosis virus oncogene (*v-rel*), a member of the rel/nuclear kappa factor B (NF-kB) family [16]. ALVs are divided into subgroups, counting from A to K, but some of them (subgroups E, F, and G) are endogenously integrated into the population genome and are vertically inherited [17]. The remaining subgroups are exogenous. Subgroups J and K were formed by the recombination of genetic material between different ALV variants in a single individual [18].

The most widespread and common variant is the ALV-J subgroup, which is genetically linked to subgroups A, B, and C and is also the most versatile, as it is found in Anseriformes and Passeriformes in addition to Galliformes [5]. The distinctive pathogenicity of ALV-J is linked with its *env* gene. The *env* gene encodes the envelope protein of ALV-J and comprises two subunits: gp85 and gp37. The gp85 protein, the viral surface’s knob-like structure, is involved in the process of viral binding and is responsible for determining the subgroup specificity of the envelope in ALV [19,20].

One particular difference between the groups is receptor usage, so co-infections of different ALV subgroups are rare [21]. Nonetheless, REV and ALV co-infections, especially ALV-J, are commonly reported as the pathogenesis of many diseases and syndromes in various species [22,23]. Current epidemiology indicates that co-infection with these viruses is more common than co-infection with REV or ALV [23]. Cohabitation of the host cell by REV and ALV simultaneously mutually accelerates the proliferation of both viruses and improves cytoskeleton protein dynamics [24]. Identical mechanisms are practiced by other viruses, such as HIV-1, which, during intensive multiplication, adjusts the host cell’s actin to assemble its own envelope particles and grow faster [25]. Co-infection of REV and ALV-J synergistically increases protein expression and accumulation of exosomal miRNAs more intensely than a single virus in mono-infection [26,27]. Regulated molecules are key regulators in pathways involved in virus-vector interaction, biological adhesion, energy metabolism, and cell growth that contribute to cancer development and progression.

Among the many regulated molecules are numerous integrins, such as integrin α3 (ITGα3), α6 (ITGα6), and β3 (ITGβ3), associated with tumorigenesis, metastasis, invasion, and cancer progression of various tissues [28,29,30]. Synergistically, REV and ALV-J inhibit cellular aging and activate epithelial-mesenchymal transition (EMT), directly inducing tumorigenesis and expanding the spectrum of cancers by offloading the NF-κB pathway in vitro [31]. Examples of miRNAs up-regulated during co-transfection include let-7, miR-1, miR-15, miR-16, and miR-151, which contribute to angiogenesis, hematopoiesis, and exocytosis [27]. One potential process of the synergistic promotion of viral replication is involved through upregulation of miR-155; suppression of PRKCI-MAPK8 pathways, key to viral replication; and TIMP3-MMP2, key regulators in viral infections.

### 2.2. Lymphoproliferative Disease Virus

LPDV is a member of the *Retroviridae* family and is closely related to REV [6]. Both viruses are oncogenic retroviruses that induce lymphoproliferation in wild and domestic birds. The initial detection of LPDV infections was observed in wild and domestic turkeys (*Meleagris gallopavo*) in the United Kingdom in 1972. The virus incorporates its RNA into the genome of an infected cell, which results in the disruption of the cell cycle and the excessive proliferation of lymphocytes. The disease presents with lymphoproliferative changes and enlargement of organs, including the spleen and liver. Additionally, tumors (lymphosarcoma) may manifest on the body of the fowl, accompanied by feather changes or weakness. This type of virus is transmitted directly through contact with other infected birds. Despite being a relatively newly discovered pathogen, its presence in wild turkey populations is increasing. Further research is required to elucidate the mechanisms of the virus’s spread and its impact on wild turkey populations [32].

### 2.3. Rous Sarcoma Virus

**Figure 1:**
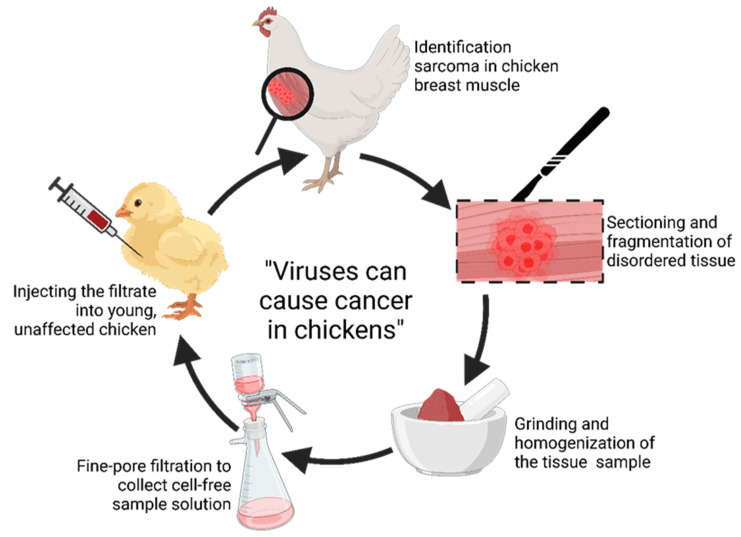
Rous’s protocol for inducing breast sarcomas in chickens using cell-free filtrate with filterable cancerous agent [33].

**Figure 2:**
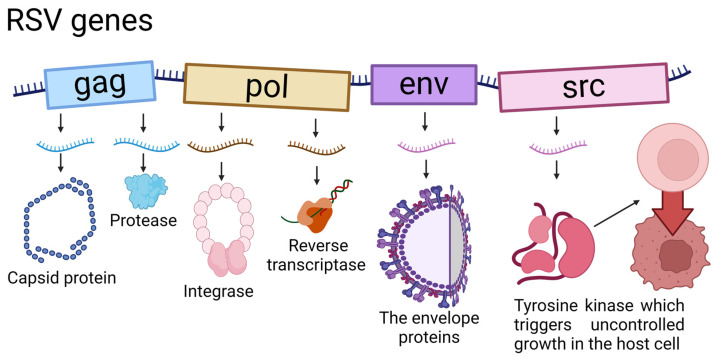
Genes organization in chicken’s RSV with factors encoded.

Rous sarcoma virus (RSV) is a retrovirus belonging to the ALV family that integrates into host DNA, causing sarcoma in chickens. Along with other avian viruses, RSV is one of the iconic viruses, although it was first described as an oncovirus by Nobel laureate Peyton Rous in 1911, whose model of research is illustrated in Figure 1 [33]. This discovery contributed significantly to the development of cancer research, extending beyond the avian model. Subsequent research on RSV contributed to two further Nobel prizes. In 1975, the prize was received for the discovery of a reverse transcriptase enzyme that is central to the mechanism of action of retroviruses [34,35]. Then, in 1989, the discovery that the viral oncogene RSV has a counterpart in host cells was highlighted, representing a breakthrough in understanding the mechanisms of oncogenesis [36]. These discoveries greatly advanced the knowledge of retroviruses and cancer development. Furthermore, they contributed to advances in human cancer research. As illustrated in Figure 2, the oncogene responsible for the oncogenic properties of RSV is the viral variant *src* gene (*v-src*). This gene encodes a tyrosine kinase, and its activation regulates many crucial signaling pathways, including PI3K-AKT, Ras-MAPK, JAK-STAT3, and FAK/Paxillin. These play a key role in cell proliferation, migration, invasion, metastasis, drug resistance, and cancer spread and progression. Although viruses are not counted as living organisms, their genome includes microRNAs (miRNAs), like the DNA of all other organisms. MiRNAs in the viral genome may play a role in regulating host gene expression and manipulating signaling pathways [37]. Viral microRNAs (v-miRNAs) are shorter and less stable than their cellular counterparts [38]. V-miRNAs are orthologs of cellular miRNAs that share a common seed sequence and thus affect the same targets [39]. Most v-miRNAs in various viruses are encoded mainly to silence pro-apoptotic genes, suppressing host cell apoptosis mechanisms [40]. The RSV sequence potentially contains five miRNAs that are synonymous with already known genes in organisms: miR-71c-5p, miR-6765-5p, miR-23a-5p, miR-4528, and miR-27b-5p [41]. Their analysis indicates that they target *Gallus gallus* tumor suppressor genes, including *TP53*, which encodes the p53 protein, a key factor in anti-cancer therapy [42], and *BRCA1*, which encodes the breast cancer susceptibility protein type 1. They reveal an important role in inhibiting breast cancer, among other things [43]. RSV-induced tumors often spread to multiple organs. This is due to the metastasis of primary sarcomas and viral inoculation [44].

The response to RSV infection can often be variable, depending on the organ involved and genetic predisposition. Individual differences depend on the haplotype of the major histocompatibility complex B (MHC-B). This affects the regression or static nature of the tumors [45]. Breeding and crossbreeding broiler chickens with the appropriate MHC-B haplotypes can enable the maintenance of an RSV-resistant flock. This significantly reduces the incidence of RSV-associated sarcomas and improves poultry production efficiency [44]. In the diversity of responses to RSV infection, individuals are described as progressors, regressors, and non-responsive chickens, in which metastatic tumors are successively enlarged, regressed, or remain unchanged. Following RSV infection, pro- and anti-inflammatory cytokines are the most prominent up-regulated molecules.

In chicks with metastasis, *IL-6* and *IL-8* were up-regulated in all organs, and *TNF-α* and *INF-γ* were down-regulated in almost all organs except the spleen. In regenerating and non-regenerating chicks, the levels were oppositely altered [46]. In addition, chemokines, innate immunity genes, and anti-tumor molecules were also significantly up-regulated, especially at the sites of the originally infected organs [47]. Expression appeared to be reversed in regressed and non-responders. Increased positive stimulation of cytokine expression occurs with secondary stimulation with the v-src antigen after prior immunization. This indicates that it is possible to obtain and use vaccines to prevent RSV infections [48]. RSV is an acutely transforming variant of the avian sarcoma and leukosis virus (ASLV). However, ASLV lacks the *src* gene responsible for encoding tyrosine kinase. Additionally, unlike RSV, it does not transform the infected fibroblasts. Two types of ASLVs can be distinguished: fast- and slow-transforming ASLVs. Fast-transforming viruses carry an oncogene, and with the exception of the majority of strains of RSV, they are all defective and thus require the presence of a helper virus in order to complete the replication cycle. These viruses induce the development of tumors in a diverse range of organs in chickens within a period of fewer than three months following initial exposure, resulting in premature death. The replication-component slow-transforming ASLVs have resulted in tumors forming in a small percentage of chickens [49].

### 2.4. Avian Myeloblastosis Virus

Avian myeloblastosis virus (AMV) is an alpharetrovirus, a member of the family *Retroviridae*, which can induce acute myeloblastic leukemia (AML) when injected in ovo or into newly born chickens [50]. Although both RSV and AMV can replicate in cultures of either embryonic fibroblasts or myelomonocytic cells, RSV has been demonstrated to induce transformation exclusively in fibroblasts, and AMV selectively transforms myelomonocytic cells in vitro [51]. Infection with AMV in hens results in a markedly elevated white blood cell count, leading to the presence of considerable quantities of viral particles in the peripheral blood [50]. Accordingly, AMV was frequently employed as a model retrovirus to investigate viral assembly and subsequently to generate vast quantities of reverse transcriptase for scientific and industrial use.

It has been demonstrated that the majority of the retroviral *env* gene of AMV has been replaced by a sequence of cellular origin. *v-myb* is a transforming oncogene that encodes the transcription factor v-Myb, which is responsible for the transformation of myelomonocytic cells.

It was observed that the *v-myb* oncogene sequences were present in both AMV and the avian E26 leukemia virus. However, they were absent in all other acutely transforming retroviruses [52]. *v-myb* is a mutated and transduced version of the *c-myb* proto-oncogene, a key player in the development of the hematopoietic system. The *c-myb* gene is crucial to a genetic switch that enables hematopoietic progenitor cells to determine their fate, such as proliferation, differentiation, or apoptosis [53]. The discovery of *v-myb* prompted the proposition that aberrant activation of vertebrate MYB may also contribute to carcinogenesis.

MYB oncoproteins are highly conserved transcription factors that are suggested to play an important role as potential therapeutic targets in human cancer. Given the essential role of MYB proteins in key cellular processes such as growth, differentiation, and survival, it seems plausible that genomic mutations or alterations in gene expression may contribute to oncogenesis. MYB has been demonstrated to positively regulate the promoters of key cell proliferation genes, including *Myc*. However, it is also capable of functioning as a transcriptional repressor. The exogenous expression of *MYB* was observed to significantly suppress the activity of several key regulators of myeloid differentiation, including *Runx1*, *Pu.1*, *Junb*, and *Cebp*. This suggests that the transcription factor employs a mechanism to suppress differentiation and promote self-renewal.

The *MYB* family comprises three members: *MYB*, *MYBL1*, and *MYBL2*, which encode the transcription factors MYB, MYBL1, and MYBL2, respectively [54]. A number of studies have identified genetic mutations and altered expression of the MYB family members in a range of cancers, including breast cancer [55], acute lymphoblastic leukemia [56], colorectal cancer [57], and clear-cell renal cell carcinoma [58].

## 3. Avian Herpesviruses

Herpesviruses have been shown in the past to be associated with oncogenesis and to be etiologic factors in some cancers of chickens as well as primates and frog neoplasms [59]. As for humans, two major oncogenic herpesviruses cause human lymphomas, namely, Kaposi’s sarcoma-associated herpesvirus (KSHV), also known as human herpesvirus 8 (HHV8), and Epstein–Barr virus (EBV) [60]. The discovery of Marek’s disease virus (MDV), the alphaherpesvirus that causes Marek’s disease, a contagious cancerous disease of chickens, came shortly after EBV was first described [61,62]. MDV shows close similarity to EBV and HSV of squirrel monkeys in terms of cell tropism and pathology [62]. Research on MDV has contributed significantly to our understanding of herpesvirus infection and latency and, crucially, has provided insight into host immune responses and the formation of virus-induced lymphomas [61].

Avian herpesviruses (AHVs) are an important group of pathogens that affect most domestic, wild, and captive bird species. With a large DNA genome that exhibits some degree of structural conservation, herpesviruses encode a range of structural and non-structural proteins that are expressed in different kinetic classes. Despite the high pathogenicity of AHV in chickens and other poultry species, there are many species for which limited information is available on pathobiological traits [63]. The pathogenesis of AHV itself can vary depending on the nature of the diseases or tissue tropisms it causes in different species, ranging from lung disease to cancer. As with mammalian herpesviruses, one of the main features of AHV is its ability to cause latent infection in different cell types of the infected host. With advances in PCR-based technologies and sequencing, more and more data on AHV phylogenetic relationships are available, which are also proving valuable for disease diagnosis. However, AHV prevention and control strategies vary depending on the pathogenesis and epidemiology of the diseases [63].

*Herpesviridae* is a family of large-DNA viruses whose natural hosts include humans, mammals, and vertebrates. Their genomes in mammals and birds show clear descent from a common ancestor. However, they have a wide range of variability in terms of nucleotide substitutions, gene content, and genome arrangement [64]. *Herpesviridae* are divided into three subfamilies, *Alpha-*, *Beta-*, and *Gammaherpesviridae*, based on their distinct characteristics and, later, on their genomic features [65]. *Gallid alphaherpesvirus 2* (GaHV-2) is a virus known as Marek’s disease virus type 1 (MDV-1) and is an oncogenic alphaherpesvirus. Alphaherpesviruses attach to cells of the exposed mucosal surface, and then enter to initiate infection. Depending on the type of cell, viral entry can occur either through the cell membrane or through endosomes after endocytosis. Alphaherpesviruses have gradually evolved different strategies to modulate host immunity to promote their fitness and pathogenesis. Although virus-infected cells can be eliminated by cell apoptosis, they can also inhibit this apoptosis by encoding anti-apoptotic virulence factors [66].

Although MD was described in 1907 by Joseph Marek, the virus (MDV) was not independently isolated in the UK and the US until 1967. The infectious particles of herpesvirus consist of more than 30 different proteins. The MDV genome is a linear, double-stranded DNA of about 175 kb in length. This genome contains about 100 open reading frames and encodes more than 70 genes, most of which have ornithologous counterparts in alphaherpesviruses [67]. Its virulent strains can maintain latent infections in their avian hosts and cause MD, a T-cell lymphoma disease. This disease has been recognized as an excellent biomedical model for studying virus-induced cancers [68]. GaHV-2 has been divided into three serotypes based on antigenic and genetic variations. Serotype 1 (prototyped by the CVI988/RISPENS strain) is most commonly used as an effective live vaccine against MD [69]. Serotype 2 (prototyped by strain SB-1) has been used for immunization in combination with MDV serotype 3 (HVT) [70]. For instance, HVT-IBD (infectious bursal disease) vector vaccine and HVT-vectored ND (Newcastle disease) vaccines are often used for in ovo vaccination of broiler chickens [71].

### Marek’s Disease

Marek’s disease virus (MDV) is the leading cause of tumors in poultry. It causes a fatal lymphoproliferative disease in chickens and causes huge losses in the poultry industry worldwide. MDV is a highly cell-associated virus because it spreads through intercellular contact. MDV causes immunosuppression, neurological disorders, chronic cachexia, blindness, and fatal T-cell lymphomas in susceptible chickens. Infection is initiated by inhaling infectious dust from a contaminated environment. In the upper respiratory tract, the infectious dust is picked up by phagocytic cells such as macrophages, dendritic cells, or B lymphocytes. These then carry the virus to lytic cells. They then transport the virus to the lymphoid organs, the thymus and spleen [72]. In these organs, the virus replicates efficiently, mainly in B and T lymphocytes, until it reaches latency. MDV enters the latency phase in lymphocytes only, but not in neurons, as most alphaherpesviruses do. At the onset of infection, the virus moves toward the skin, especially into the feather follicles. MDV primarily causes latent infection of CD4+ T lymphocytes, which can transform, causing the development of lymphomas. Infected cells then transport the virus to the epithelium of the feather follicles, where an infectious, cell-free virus is produced and spreads in the environment for 2 weeks after infection [73]. MDV strains have been divided into three serotypes based on antigenic differences and biological characteristics. Strains classified as oncogenic are classified as serotype 1, while non-oncogenic strains isolated from chickens and turkeys are classified as serotypes 2 and 3 [65]. Authenticated serotype 1 viruses are used as vaccines, which provide better protection and induce a better immune response [63].

Live MD vaccines prevent partial paralysis and lymphoma formation. They also mediate the reduction in virus replication and assembly in FFE but do not prevent host infection, virus replication, and shedding [74]. However, despite the control of Marek’s disease since the 1970s, the underlying mechanism is not clear. Unlike most animal vaccines, as well as human vaccines that require the activation and engagement of the acquired immune system, MD vaccines induce partial protection as early as 24–48 h after vaccination. The acquired immunity depends on the protective efficacy of a particular vaccine; it can reach 95–100% at 5 days after vaccination [75].

Despite control of the disease through vaccination with attenuated or non-pathogenic MDV strains, vaccine failures do occur. This may be attributed to the fact that field strains are undergoing constant evolution, resulting in the emergence of pathotypes with enhanced virulence. However, the most prevalent cause of failure is the administration of vaccines in an erroneous manner. Two strategies for enhancing the efficacy of vaccination programs are deemed acceptable: the development of novel vaccines and the optimization of existing vaccines. This requires investigating the optimal time and route of vaccine administration and optimal vaccination schedules for chicks of different breeds. The accurate quantification of MD vaccine virus in vaccinated chicks will contribute significantly to the understanding of vaccine protection [76].

## 4. Importance of Avian Oncoviruses for Biomedical Research

Studies by Oluf Bang and Vilhelm Ellerman at the University of Copenhagen in 1908 showed that ALV was transmitted between chickens by cell-free filtration and could then lead to leukemia [77]. Furthermore, Peyton Rous at Rockefeller University in 1910–1911 demonstrated that solid tumors could arise from viral infections in chickens, extending the earlier findings of Bang and Ellerman to demonstrate cell-free transmission of solid tumor sarcoma in chickens. The virus is now known as Rous sarcoma virus [78]. However, it was not noted that this was the first successful transmission of a naturally occurring tumor, as leukemia was not yet considered a cancer at the time. Many people objected to the validity of these findings because infectious bird tumors were not accepted as accurate models of cancer in humans [79]. Moreover, the importance of this study was not fully appreciated until the discovery that mouse leukemia could be induced by viruses [2]. In the early 1950s, it was understood that viruses could cause cancer by inserting new genes into the genome of host cells. The use of the term “oncovirus” was popularized a little later, in research on acute transformation retroviruses in the 1950s–1960s [80]. Research on avian oncoviruses has already provided very important information on viral mechanisms of human cancer; avian oncoviruses have been useful not only in epidemiology, but also in studying cell cycle control mechanisms relevant to the role of tumorigenesis, such as the retinoblastoma protein [81].

**Figure 3:**
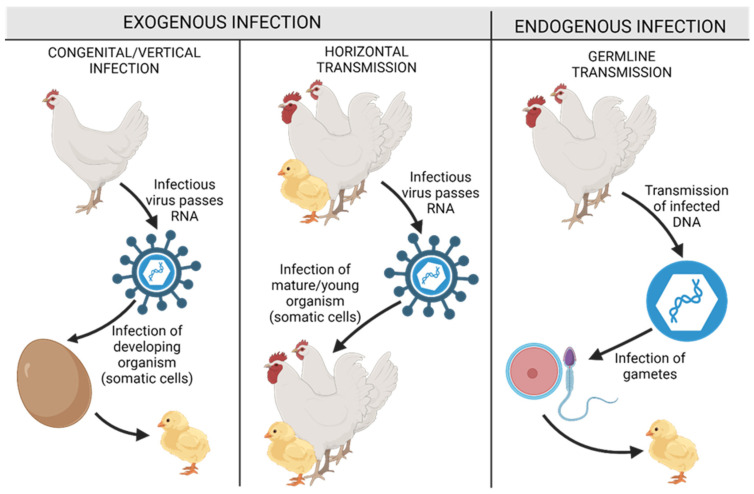
Avian leukosis viruses’ DNA transmission methods, including possible hosts and targets. The figure illustrates two distinct types of infections in chickens: exogenous infection (**left**) and endogenous infection (**right**). ALV may be transmitted vertically through congenital or egg contamination and horizontally through contact. In the vertical transmission pathway, contamination of the oviduct by the virus leads to the infection of embryos during their development. Moreover, ALV may be transmitted in the germline across generations (endogenous route).

The initial belief in an infectious etiology of cancer emerged in classical times, prompted by reports of “cancer houses” in which many residents contracted a particular cancer, as well as reports that married couples sometimes contracted similar types of cancer. Furthermore, cancer in many cases appeared to be transmitted vertically from mother to child, as shown in Figure 3 [82]. Despite this widespread belief, significant research on this hypothesis in the 19th century was unsuccessful in demonstrating a carcinogenic role for fungi, parasites, or bacteria, leading to the demise of this belief. However, a small number of researchers have maintained that the failure to detect an infectious carcinogen does not necessarily rule out the general idea of the infectious nature of cancer and instead supports the view that the causative organism has simply not yet been identified, as smaller entities, undetectable by standard microscopy, may indeed be malignant agents [83].

The first hypothesis that cancer is transmitted by a non-cellular agent was supported by studies by M’Fadyan and Hobday [84], who demonstrated the cell-free transmission of canine oral warts using cell-free extracts. Similarly, Ciuffo conducted comparable studies on the transmission of human warts in 1907 [85]. The significance of these discoveries has not been fully appreciated because warts are benign hyperplasias, not malignant tumors. Over the last decades, several additional animal oncogenic viruses have been identified, and Rous was eventually awarded the Nobel Prize in 1966 for his groundbreaking work [83]. Rous’s work arguably was the basis for Richard Nixon’s decision to sign the National Cancer Act in 1971 [86], which undoubtedly shaped the future of oncovirus research. Viruses are now recognized as the true causes of many human cancers, and it is estimated that 15 to 20% of all human cancers may have a viral etiology [2].

The WHO International Agency for Research on Cancer found in 2002 that 17.8% of human cancers arose from infection, and 11.9% of them were caused by one of the seven viruses listed [87]. A separate study of more than 2600 samples from 38 different cancers in 2020 found that 16% of them originated from a virus [88]. Such viral cancers can be prevented by vaccination or better managed by earlier detection with simple blood tests and treatment with antiviral agents. Therefore, further research on avian retroviruses is crucial for managing human cancer pathology [89].

## 5. The Role of Chicken Inbred Lines in Advancing Cancer Research

In cancer research, chicken inbred lines have been used for many years, playing a significant role in advancing oncology. Inbred lines are breeding lines formed by crossing related individuals over several generations. The resulting individuals are homozygous, which means they possess two identical copies of each gene. In hen breeding, these lines are created to control genetic variation as well as to develop characteristic traits such as disease resistance, body weight control, and increased egg production. Inbred lines of hens are particularly important for understanding the genetics of resistance to infectious diseases [90].

Key discoveries involved RSV, which can cause cancer in hens. This research has greatly contributed to understanding oncogenic viruses in humans, as retroviruses can integrate their DNA into the host’s genome, leading to neoplastic transformation. Studies on homozygous inbred hens helped scientists uncover how viruses disrupt host genes, a critical discovery that paved the way for identifying similar mechanisms of viral oncogenesis in humans, particularly for retroviruses [77]. Furthermore, studies on inbred hens led to the discovery of proto-oncogenes, which can mutate or become abnormally activated, transforming them into oncogenes and ultimately leading to tumor formation. The identification of proto-oncogenes in inbred lines was groundbreaking for the understanding of human oncogenesis, as the same genes have been shown to regulate cell growth in humans.

Moreover, mutations in these genes have been associated with various human cancers, including colorectal, lung, and breast cancer [91]. Additionally, due to the stable and consistent genetic profile of hen inbred lines, scientists have been able to investigate the role of immune system genes, such as the major histocompatibility complex (MHC), in cancer resistance [92]. Research in hens has demonstrated that certain inbred lines have differential susceptibility to cancer depending on their MHC gene variants, which has also been shown to be important in humans. Hen models have also contributed to the understanding of the role of tumor suppressor genes such as p53 [93].

## 6. Laying Hens as a Model for Ovarian Cancer Research

Ovarian cancer (OC) is a deadly gynecologic malignancy that poses numerous challenges for researchers trying to understand its origins and develop effective treatments. Recognition of OC’s molecular pathogenesis and heterogeneity offers new opportunities to develop early detection strategies and effective therapies. Higher-order animal models enable the reconstruction of the tumor architecture and microenvironment. Such models have expanded our knowledge of the genetic events that initiate tumorigenesis and the cells of origin of ovarian tumors. The rodent model has been employed in a plethora of studies to investigate the development and progression of OC. However, this model has inherent limitations, including the fact that OC does not develop spontaneously in rodents and that induced carcinomas differ from spontaneous OC in humans [94,95]. Indeed, mouse models have generated some information on OC development at the molecular level. However, the difficulty in transferring this information into the clinic is a significant challenge.

OC develops spontaneously in laying hens, providing an opportunity to study genetic, biochemical, and environmental risk factors as well as tumor initiation, progression, and histological origin [96]. These tumors closely mimic the histopathological features and progression of human ovarian cancer, making hens a suitable model for studying this disease [97]. In contrast to the limitations of artificially created rodent models, biomarker discovery studies in chickens allow for comprehensive investigation through the use of matched longitudinal plasma samples and tissue biospecimens, which can be harvested for genomic, proteomic, or metabolomic signatures [97].

Despite the differences in the anatomy of the two species, there are many similarities in the context of OC development. The domestic hen is subjected to almost daily ovulation for at least two years of egg production, which, firstly, leads to a shorter lifespan and, secondly, to a higher incidence of OC. This is somewhat analogous to humans—an increased incidence of OC can now be observed in developed countries due to continuous ovulation without fertilization. The hen model supports the hypothesis of continuous ovulation in the development of ovarian cancer [98]. Similar to humans, the incidence of OC in hens increases with age. The incidence of OC in 3–4-year-old hens is estimated to be between 10% and 20%, with an observed increase to 40% as the birds approach 6 years of age [97,99]. However, some have found that due to the absence of the luteal phase in hens, the lack of progesterone may be the reason for the increased cancer risk [100]. Additionally, the hen does not menstruate [101].

It has been observed that there are notable similarities in the morphology and histopathological presentation of ovarian tumors in humans and chickens. Similarly to the condition observed in women, OC in hens is characterized by the formation of a solid tumor mass in the ovary, with moderate to profuse ascites when the disease has metastasized to other distal organs [102]. Similarities in the histological types of epithelial ovarian carcinomas have been observed in laying hens, as is the case in humans. The most frequently observed tumor types in hens are serous and endometrioid epithelial ovarian carcinomas, with sporadic incidence of other carcinoma types [103]. At the molecular and cellular levels, hen ovaries are also similar to those of humans. They exhibit the same molecular markers, including mucin 16 (MUC16 or CA125), selenium-binding protein 1 (SELENBP1), and cyclooxygenase 1 (COX-1) [104,105].

Furthermore, it is noteworthy that the hen’s oviducts exhibit a striking resemblance to the human condition. In addition, the frass performs analogous functions, such as capturing ovulatory eggs, and serves as a fertilization site. The cloaca of the hen also allows for exposure of the oviductal tufts to the external environment, thereby increasing the risk of infection by pathogens [105]. Similarities between hens and humans can also be observed in the context of OC pathology. Studies on laying hens suggest the importance of the oviduct epithelium in the development of spontaneous ovarian cancer, which is similar to that in humans [101]. Moreover, ovarian adenocarcinoma shows similarities with regard to histological structure [103].

**Figure 4:**
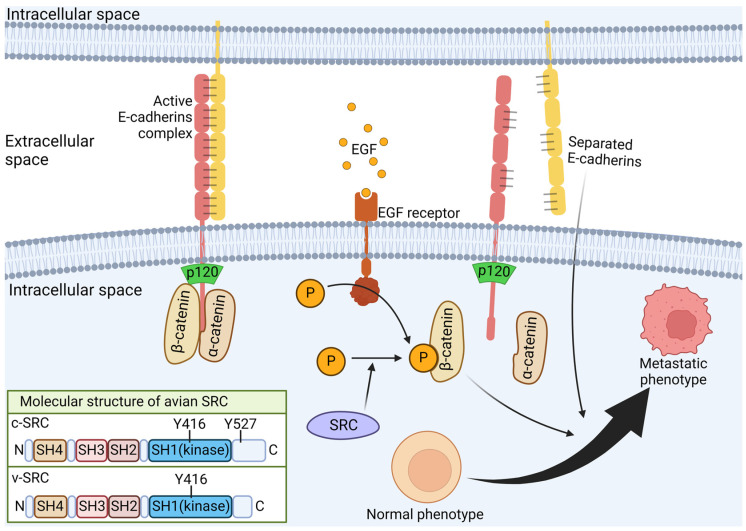
Significance of the E-cadherins and their signaling pathway in tumorigenesis.

In addition, the development of ascites and peritoneal metastases resembles the pathogenesis of advanced metastatic OC in humans [106]. Several markers, including CA125, p53, or E-cadherin, show analogous trends in terms of expression levels [104,107,108]. Because of its several advantages, the model can be used not only for the preliminary evaluation of possible therapeutic/chemopreventive agents, but also to characterize the molecular mechanisms governing the disease. A study conducted in 2020 showed an important role for the miR-200 family in the development of inclusion cysts that may underlie spontaneous ovarian cancer formation, among others, in a chicken model, demonstrating not only the up-regulation of miR-200 family members but also of E-cadherin, fulfilling the function of a cell–cell adhesive factor, with high tumorigenesis significance, as shown in Figure 4 [109]. A further study aimed to expand the limited understanding of how selenium-binding protein 1 (SBP1) expression operates in OCs. This study revealed that SBP1 is expressed in both normal ovaries and ovarian tumors. However, qRT-PCR demonstrated decreased mRNA expression in 80% of ovarian tumors [110].

A study by Ansenberger et al. examined the expression of E-cadherin in the hen ovary and compared it with that in human OC. Western blot and qRT-PCR experiments revealed a significant increase in mRNA and protein expression of E-cadherin in the ovaries of cancerous hens compared with those of non-pathogenic hens. E-cadherin is an adherent protein whose expression is reduced in numerous cancers. However, in primary human OC, its expression is increased. Moreover, the study showed that comparable levels of E-cadherin expression were observed using IHC in both human and chicken OC tissues, and similar E-cadherin expression was also found in primary ovarian tumors and peritoneal metastatic tissues from cancerous hens [107].

These findings suggest that the up-regulation of E-cadherin represents a pivotal early event in OC formation, potentially exerting a significant influence on the initial development of not only primary ovarian tumors but also secondary tumors within the peritoneal cavity. These observations indicate that E-cadherin represents a crucial target for the prevention of metastatic ovarian cancer (OC), thereby substantiating the suitability of the laying hen as a model for the study of human epithelial ovarian carcinoma [107].

Another advantage of the chicken as a model for studying OC in women is its exposure to environmental factors. An analysis of the effects of these factors on the risk of OC in hens may provide important information on their role in the development of OC in humans [111]. Since ovarian carcinogenesis in laying hens is spontaneous, it is possible to evaluate chemopreventive compounds for the treatment of OC in this model [112].

Administration of medroxyprogesterone acetate to laying hens confirmed that reducing ovulatory events can serve to prevent ovarian adenocarcinoma and that this avian model is highly suitable for OC research [113]. Furthermore, a study conducted in 2018 on laying hens investigated the chemopreventive properties of lycopene, in particular, its molecular mechanisms of action on OC. It was observed that lycopene supplementation significantly reduced tumor incidence, number, and size. These results suggest that the chemopreventive effect of lycopene on OC is mediated through antioxidant and anti-inflammatory mechanisms [114]. Another example is genistein, the main isoflavone present in soybeans, which is said to provide anti-oncogenic effects against many types of cancer, including OC. A recent study demonstrated the preventive effects and mechanisms of genistein in a laying hen model. The study observed that genistein supplementation resulted in a significant reduction in the incidence, number, and size of ovarian tumors. The findings of this study strongly suggest the potential of genistein in ovarian cancer chemoprevention and serve to highlight the effects of this substance on the molecular pathways involved in spontaneous ovarian tumorigenesis [115]. Another study evaluated the effects of two flaxseed dietary components, ligand and omega 3 fatty acids, on estrogen metabolism and other processes in a model of ovarian cancer in laying hens. The study showed that these compounds exert anti-cancer effects by regulating the levels of 2-methoxyestriadol as well as SMAD7, which are involved in the methoxyestradiol-mediated apoptosis pathway. In addition, p38 and ERK1/2 MAPK activation was demonstrated [116].

Although the laying hen model presents a promising in vivo system for investigating the mechanisms underlying OC initiation and progression, it is not without certain limitations. In contrast to the extensive research on mouse models, where transgenesis is well established, the availability of transgenic chicken models is restricted. This hinders the investigation of specific genes and their roles in the development of OC in hens. In addition, although new methods are being researched, at this point genetic manipulation in hens is more complicated than in mice. Nevertheless, the laying hen model is of particular value for the evaluation of chemoprevention strategies and the testing of natural or pharmacological agents that may potentially prevent the development of ovarian cancer in humans. Thus, the OC model provides the possibility of large-scale drug screenings and the evaluation of drug efficiency in a robust model.

Current research is focused on exploring new strategies for the genetic manipulation of OC hen models. Advances in genome-editing technologies have significantly enhanced the development of avian species as models, enabling studies not only on disease resistance but also on pharmaceutical applications. Genome-editing tools, such as the clustered regularly interspaced short palindromic repeats (CRISPR)/Cas9 system, have been successfully introduced into early embryos across various animal species. The CRISPR/Cas9 system represents a significant advance in the field of gene editing. It comprises two sets of RNA, 20 bp CRISPR RNA (crRNA) and universal trans-activating crRNA (tracrRNA), as well as a nuclease derived from Streptococcus pyogenes type II, the Cas9 protein (Cas9), which induces the cleavage of the target loci [117]. For instance, CRISPR/Cas9 was employed to disrupt the recombination-activating gene 1 (RAG1), producing chickens that lack mature B and T cells. These genetically modified chickens serve as a tool for studying various lymphocyte populations in the absence of B and T cells and for investigating a range of diseases, including ovarian cancer [118]. However, in birds, a more dependable method for creating genome-edited models involves introducing the CRISPR system into primordial germ cells (PGCs), which are stem cells capable of contributing to the germline. In 2016, Oishi et al. described a method for efficiently generating ovomucoid gene-targeted chickens using the CRISPR/Cas9 system. Their approach involved transferring temporarily drug-selected PGCs into recipient embryos, which were irradiated with gamma rays to remove the embryos’ own PGCs [119].

In subsequent studies, Oishi et al. employed the CRISPR/Cas9 system to facilitate the integration of the human interferon beta (hIFN-β) gene into the chicken ovalbumin gene, specifically, into exon 2 [120]. Furthermore, the application of CRISPR/Cas9-mediated genome editing in avian species holds great significance in this area of research.

The study of Rieblinger et al. successfully generated transgenic chickens that express Cas9 across all organs while maintaining normal health and fertility. Cas9 functionality was confirmed by targeted gene disruption in lymphocytes, brain tissue, and precise DNA excision in the heart. Using phiC31 integrase-mediated integration of an SpCas9 expression construct, Cas9 was integrated into the chicken genomes, and the editing efficiency was validated by a 17% reduction in EGFP fluorescence. These Cas9 transgenic animals provide a valuable resource for genome editing in agricultural and biomedical research, supporting reverse genetics and cross-species comparisons in genomics and disease modeling [121].

A further area that has yet to be explored is the identification of genetic susceptibility to OC in hens. Genome-wide association studies (GWASs) hold the potential to uncover critical genetic markers, facilitating the selection of specific populations for more focused investigations. Moreover, targeted research on well-established susceptibility genes such as *BRCA1* [122] and *BRCA2* [123], which have also been identified in chickens, could provide additional evidence supporting the validity of the chicken as a model for OC studies. Furthermore, the recent genome sequencing of the chicken will facilitate the application of the hen model in the investigation of gene therapies for ovarian cancer.

In conclusion, the similarities between hen and human OC with a precisely regulated genetic background offer an exceptional experimental model for investigating the initial stages and progression of spontaneous OC in a controlled environmental setting. The epidemiological and molecular features provide strong evidence that the laying hens are a suitable model, replicating many key characteristics of human ovarian cancer. Maintaining hens is a relatively easy and cost-effective process, and their rapid ovulatory cycle and susceptibility to disease make them an ideal subject for larger-scale research projects.

## 7. The Avian Embryo as a Model of Carcinogenesis

An understanding of chick embryo development has significantly contributed to the broadening of knowledge regarding cancer processes. Several mechanisms that govern cell survival and migration are shared between developmental programs, oncogenesis, and metastasis [113]. The chick embryo is one of the models of epithelial-to-mesenchymal transition, a process associated with both development and metastasis. For instance, Prakash et al. have revealed that upregulation of ribosome biogenesis in G1/S arrest facilitates EMT in several species, including in chicks, and concluded that these events are, therefore, linked further to differentiation and metastasis [124].

**Figure 5:**
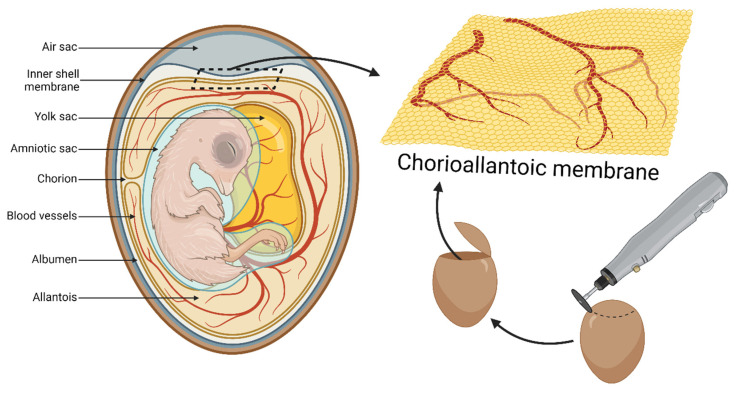
Anatomy of the chicken embryo at the 14th day of development with highlighted chorioallantoic membrane (CAM) localization and sourcing.

**Figure 6:**
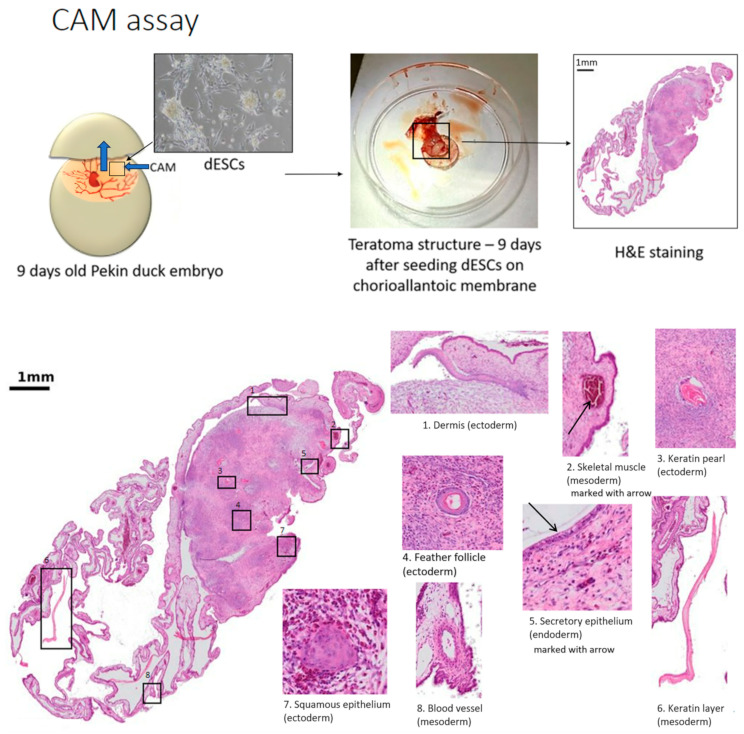
Molecular structure of the chorioallantoic membrane (CAM) and imaging of differentiating cells seeded on the CAM, forming a teratoma. Tissues were stained using hematoxylin and eosin.

The chick embryo is a highly adequate in vivo model due to the accessibility of the chorioallantoic membrane (CAM), the structure shown in Figure 5 and Figure 6. Since the embryo is immunodeficient, this well-vascularized extraembryonic tissue can be easily used for xenografting both cancerous (primary tumors and cell lines) and healthy tissues [125]. CAM can reflect cancer hallmarks such as extensive growth, tumor vasculature formation, or remodeling of the microenvironment, as well as invasive capacities [126]. It served in basic cancer research focused on oncogenesis and metastasis, starting with Rous’s study in 1911 [33,127,128]. A more recent study by Deryugina et al. demonstrated that intravasation occurs mainly in the core of the primary tumor, as opposed to the conventional model. This suggests that EGFR is crucial for the development of the vasculature essential for this process [129]. Additionally, the rich vasculature of extraembryonic tissues and the embryo itself contributed to early descriptions of tumor-induced angiogenesis [130].

CAM assay can become a new standard in studies of vasculature development and the molecular mechanisms underlying it as well as in the assessment of potential angiogenic and anti-angiogenic compounds [131]. The development of advanced imaging techniques, including in real time, enables visualization of changes in the CAM vasculature, for example, in different developmental stages or under the influence of tumor tissue introduction. The state of the art has been reviewed elsewhere [132]. CAM assay can also serve as a bridge between in vitro and in vivo studies in high-throughput screening of potential anti-cancer compounds [133,134]. As visualization of CAM vasculature is easier than of mammalian, it is a far more convenient model in the assessment of the potential anti-cancer action of compounds as well as their localization in the tumor and surrounding microenvironment [113]. For example, Cao et al. screened a library of FDA-approved drugs and demonstrated in an in vivo chick embryo CAM model that decylubiquinone (DUb), a coenzyme Q10 analog, is a potential tumor angiogenesis suppressor [135].

The development of personalized therapies is now a crucially important issue in cancer research. For now, an effective, easy-to-maintain, short-term preclinical in vivo patient-derived xenograft model, which would improve this development, is lacking. A group led by Delloye-Bourgeois has created an avian embryonic model of neuroblastoma, in which cancer cells were grafted into the embryonic tissues to better mimic the tumor microenvironment. An appropriate NB model was developed that captured both the early stages and the disseminated form of the disease. Their study provided insight into the molecular background of NB metastatic dissemination—it demonstrated that downregulation of SEMA3C is a switch for cell detachment and subsequent metastasis [136]. In 2021, the researchers also generated a new in vivo triple-negative breast cancer model of this kind and proved its effectiveness in addressing various preclinical questions [137].

Obviously, despite the many advantages of this model, there remain some limitations. In the CAM assay, tumor cells do not usually form macroscopic metastatic colonies as there is only a short time until hatching [131]. It is also difficult to distinguish between neovascularization and rearrangement of already present vessels [134]. Additionally, although the embryo is usually considered immunodeficient due to the immaturity of the immune system, inflammatory reactions are present if the experiment is conducted for more than 15 days [138]. CAM is also sensitive to changes in the experimental conditions, including pH, osmolarity, or level of keratinization [139].

## 8. Conclusions

The avian model will continue to be an important model system to understand cancer biology. The extensive use of poultry in food sourcing has driven the need to delve deeper into the diseases developing in the avian model. Despite a relatively distant evolutionary relationship, the bird model shows similarities in tumorigenesis induction or progression, consisting of a valuable source of research knowledge about carcinogenesis affecting the human species. Since the discovery of the first oncovirus, research on the avian model has developed and progressed in parallel with research on the human model. The discovery of viral cancers made it possible to adequately describe and understand the processes of viral carcinogenesis and, above all, to create tools, such as vaccines, that prevent tumor induction. The avian model as a research tool has been instrumental in elucidating the core mechanisms of tumorigenesis, metastasis, and drug response as well as the impact of specific genes, signaling pathways, and environmental factors on cancer development and progression. All this research has contributed to decreasing the epidemiology of viral-caused cancers. Currently, oncoviruses that have human variants are described. One such example is MSV. In addition to oncoviruses, bird tissues are a valuable model for various cancers, including ovarian cancer. The laying hen is the best-known model of spontaneous ovarian cancer development, with a histological picture similar to that of humans. Replacing a human with a laying hen model allows for a much more efficient and safer research model of ovarian cancer, which has already helped to increase the chances and prognosis of treatment. Additionally, the avian embryo provides a favorable environment for the real-time observation of tumor growth and metastasis, facilitating the visualization of dynamic cellular processes. The rapid development of the embryo enables high-throughput research on various strategies and covering various structures.

The avian model has contributed widely to human healthcare, but the constantly evolving state of knowledge points to further requirements and limitations of the various currently used models. The universally applied cellular research model does not follow all the requirements of developing tissue, so the further use of an avian model could increase the chances of enhancing knowledge about human carcinogenesis.
